# Tea trolley teaching in critical care: Integrating evidence‐based practice with library services

**DOI:** 10.1111/nicc.13264

**Published:** 2025-02-02

**Authors:** H. McGivern, S. Bridge, S. Sutherland, E. Reynolds, J. Ede

**Affiliations:** ^1^ Bodleian Health Care Libraries University of Oxford Oxford UK; ^2^ Oxford University Hospital NHS Foundation Trust Oxford UK; ^3^ Oxford Institute of Applied Health Research Oxford Brookes University Oxford UK

**Keywords:** critical care, health services research, leadership, research implementation, research in practice

## Abstract

Tea trolley teaching is a tried and tested method of providing bedside education to hospital staff. This project aimed to integrate the tea trolley teaching model, already established in our local critical care unit, with library services. The goal was to equip clinical staff with the necessary training to retrieve literature and support evidence‐based practice. Our evaluation highlights the value of this combined intervention of teaching research skills to upskill staff working in our intensive care units. This paper describes a scalable model of critical care bedside education that integrates library‐focused teaching to upskill nurses in some of the prerequisite skills needed for evidence‐based practice (EBP).

**What is known about the topic**

- There is an increasing need for critical care staff to remain up‐to‐date with an expanding knowledge base.
- A high turnover of staff and an increasingly junior critical care workforce creates additional education requirements.
- Librarians and the library service add significant value to evidence‐based practice (EBP).

**What this paper adds**

- The tea trolley teaching model is an effective way to promote library‐focused teaching and staff engagement within critical care.
- Education requirements of critical care staff can be addressed through establishing strong relationships with library services and clinical librarians.
- The tea trolley teaching model addressed one of the key elements within the Iowa EBP framework and can be adapted to other clinical areas to engage other members of the nursing workforce in developing their research skills.

## 1 BACKGROUND AND RATIONALE

Critical care nursing is a highly complex and skilled branch of general nursing. Hospital inpatients admitted to an intensive care unit (ICU) are acutely unwell and require specialised treatments and interventions such as ventilation, renal replacement therapy and advanced cardiovascular monitoring.^1^ These patients have increasingly complex needs, greater numbers of comorbidities and polypharmacy requirements.^2^ The national critical care workforce is also experiencing high staff turnover rates with up to one in two critical care nurses expecting to leave their current role within 3 years.^3^ This attrition will result in a junior workforce that requires additional educational support to deliver good quality care to patients. An expanding nursing curriculum and fewer experienced critical care nurses creates a greater demand for staff that can both retrieve and examine literature or evidence to support and inform patient care and EBP.^4^, ^5^

The Iowa Model of EBP highlights the importance of reviewing and critiquing literature as a key component^6^ and librarians can support clinical staff to develop these research skills. Conceptually, librarians visiting a clinical area is not novel. Librarians are reported to have participated in ward rounds for several decades,^7^, ^8^ including in our own hospital.^9^ One NHS hospital piloted integrating a librarian into their critical care team to attend ward rounds and search for relevant evidence, as well as supporting educational activities with a grant from NHS England.^7^, ^10^ While this approach has shown promising results,^7^ it requires financial investment and does not focus on upskilling nursing staff in critical care.

### 1.1 Project aim

We aim to describe our quality improvement (QI) project that integrated library‐focused teaching within a pre‐existing local tea trolley education programme and describe the impact of this intervention as perceived by critical care staff.

## 2 METHODS

This quality improvement project utilised the Plan, Do, Study, Act (PDSA) cycle as a framework to test out the idea.^11^, ^12^ The ‘Plan’ and ‘Do’ phases relate to the planning and where and who was going to carry out the intervention, as well as what the intervention was going to look like. The results describe the ‘Study’ phase and the ‘Act’ phase, the conclusion, describes reflections on improving the intervention based on the results.

### 2.1 Service

The intervention was developed and implemented within an NHS Trust that constitutes three tertiary referral hospitals and one smaller hospital with 1500 beds and serves a population of over 600 000. The general, multilevel adult ICU consists of two units across two sites and has a critical care bed capacity of 48.

### 2.2 Intervention

#### 2.2.1 PDSA phase: Plan

Previously, the tea trolley teaching model has demonstrated impact through building confidence in practical skills that are expected of staff working on the wards, refreshing knowledge of existing guidelines and the rapid dissemination of new protocols and information to staff.^13^, ^14^, ^15^, ^16^, ^17^, ^18^, ^19^

A tea trolley teaching programme was implemented in our ICU in September 2022. Since its initiation locally, our tea trolley teaching programme has covered a range of topics from anaphylaxis to medication management and has been delivered by different members of the team. The QI initiative was registered with the Trust's clinical governance system and allocated a Ulysses Number (ID 9562). The primary objective of a tea trolley teaching session is to provide small group bite‐sized education with several short take‐home messages brought to staff working in ICU during their shift.^13^, ^14^, ^15^, ^16^, ^18^, ^19^ With a high number of new and junior staff in the local ICU, this approach enabled the delivery of education to clinical staff without taking them away from their clinical responsibilities.

A training need identified by senior nursing staff in the local ICU was the prerequisite research skills for EBP. To make best use of the available evidence, clinical staff need to be able to find the existing evidence. Nurses most frequently turn to their peers or general search engines for information, and many are not confident in the use of bibliographic databases or point‐of‐care tools such as PubMed and BMJ Best Practice.^4^, ^20^

The library is well placed to support the development of the skills needed to find high quality evidence. In our context, library support for the critical care team was historically supplied via email requests, ad hoc one‐to‐one training sessions or as part of formal academic courses. This service is well‐liked and valued by those who make use of it; however, we recognised that it was challenging for some clinical staff to find time away from the ward to make use of such support. With the existing tea trolley teaching programme in place, we saw an opportunity to bring library‐led EBP training into the ICU. Our intervention sought to use the existing tea trolley teaching education strategy to raise awareness of the support librarians can provide for EBP, deliver ad hoc training and crucially help librarians build a relationship with clinical staff.

#### 2.2.2 PDSA phase: Do

**FIGURE 1:**
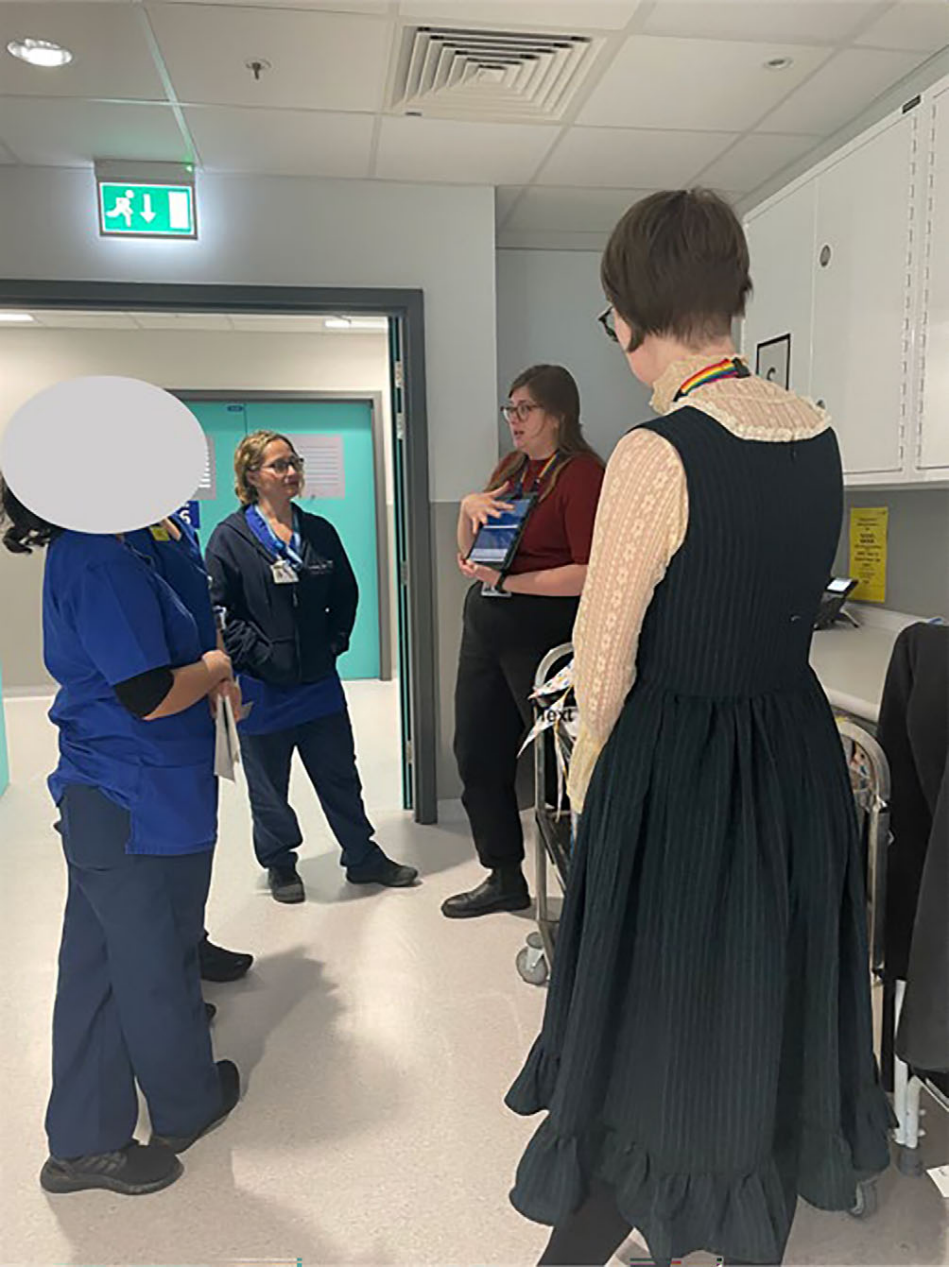
Librarians and divisional research lead leading a tea trolley teaching session in the intensive care unit (photo with permission).

Librarians and clinical research staff moved around the ICU with a trolley holding tea, coffee and biscuits, as well as learning materials and guides. The trolley was stationed in conveniently accessible areas of the wards where it would not cause any obstruction. The tea trolley would remain stationary for 10–15 minutes while the librarians and divisional research lead conversed with clinical staff (Figure 1), before moving to a new part of the ward.

### 2.3 Evaluation

Each teaching session was evaluated through qualitative facilitator reflections/field notes which details the content of each session and the questions that were raised by staff. The challenges encountered when delivering the education session were also captured within the field notes data. Survey responses from each staff member who attended were submitted at the end of each session by means of a quick response (QR) code. Survey Likert data were descriptively analysed calculating proportions and percentages.

## 3 RESULTS

The library‐focused tea trolley teaching sessions (*n* = 9) were administered between January and July 2024 to a total of *n* = 45 staff (mean = 5 ± 2.1 per session). These sessions occurred between 3 and 4 PM which was deemed a period of lower workload, to maximise the ability of clinical staff to attend. Additionally, these sessions were scheduled in advance with managers, physiotherapists, pharmacists, doctors, deputy sisters, clinical educators and researchers, to build upon interpersonal skills across the different roles within the unit, as evidenced previously.^13^, ^18^, ^19^

The librarians opened with an overview of the support and resources available, but the remainder of the session was loosely structured and responsive to accommodate the training needs and different learning styles of the staff in attendance.^13^, ^14^ This often included demonstrations of BMJ Best Practice, for example. Most nurses had not used the BMJ Best Practice App before, and this generated a lot of interest as a source of quick and reliable information. The most common questions from attendees were about gaining access to full‐text journal articles and joining the library. These queries could be addressed at once, as library card registration forms and step‐by‐step guides for creating an OpenAthens account were also stored on the tea trolley. As such, the number of OpenAthens accounts created totalled 27, and were directly attributed to these sessions. During one session, attendees were interested in reference management software, so a short ad hoc training session on RefWorks was provided.

It became clear that it would be more effective to teach and demonstrate in smaller groups. Conversations with attendees focused on their academic experiences and finding out about the courses and career development opportunities they hoped to participate in. This gave rise to some interesting conversations about potential barriers to further study or starting QI projects, with several staff expressing concerns about finding evidence and then critically appraising it. The librarians discussed these concerns and provided detailed information about the support offered. The number of staff that proceeded to request longer and more tailored, one‐to‐one support from the library for their course assignments or QI projects rose to five. Furthermore, a divisional research lead provided information about her role and explained the support on offer for academic writing and research methods. We felt that bringing together these resources was highly beneficial and seemed to allay some of the doubts expressed by nurses about their ability to begin an academic course or QI project.

More than 73% of attendees agreed that the teaching was engaging, informative, pitched at the right level and for the right duration. Furthermore, 90% of staff who attended strongly agreed that what they had learnt during these sessions would be useful to them in the future. More than 71% of attendees enjoyed the tea trolley teaching sessions and found the sessions interactive. Although, 73% reported that there were no barriers to accessing a tea trolley teaching session, limitations because of busy shifts and the workload experienced in ICU, such as patient distress, machine alarms and noise, did impact the staff learning experience occasionally. To help mitigate this and ensure that patient care was not compromised, a senior clinician would take over patient care to enable staff teaching.

## 4 CONCLUSION

Utilising the tea trolley teaching model to integrate a library‐focused education topic was both feasible and effective. It was well‐received by critical care staff, as evidenced by high levels of engagement, satisfaction and interest in resources such as BMJ Best Practice and access to full‐text journal articles. This adaptable teaching method also offers the flexibility to incorporate other EBP elements. For instance, future iterations could align with the Iowa Model of EBP, addressing components like problem identification, question formulation or standardised evaluation outcomes.^6^

Locally, an area of focus for our critical care team is ensuring nurses have the skills and confidence to successfully apply for ICU courses as well as increase research capacity and capability within ICU. With this in mind, we are developing a bundle of resources to support staff applying for ICU courses and intend to signpost this information during tea trolley sessions. Furthermore, a workshop is being designed to cover critical appraisals of peer‐reviewed journal articles, as this is a pre‐course requirement. These strategies address some of the challenges in critical care education outlined, including the intake and success rate of course applicants. We do, however, acknowledge that there is a limit to how much research skills training can be provided within the bite‐size sessions of the tea trolley teaching format.

After the success of the sessions in the ICU, consideration has been given to how we might expand in‐ward library training to support skills development for some elements of EBP in areas without an established tea trolley teaching programme. A variation of the library services tea trolley teaching session has been trialled in two additional clinical areas; these sessions were well attended, and anecdotal evidence suggests they were well‐received by staff. However, the prospects for expanding this style of training are dependent on several factors. This includes staff engagement, interdepartmental collaboration spearheaded by clinical research leads or a designated team member who can co‐ordinate arrangements for tea trolley teaching and invite librarians to be present on the wards at a time that is convenient for hospital staff.

Our local improvement work suggests that other clinical areas should consider utilising and fostering links with their local health care library and librarians. These relationships can provide additional support to continually upskill the junior workforce in the ICU, and across all clinical areas. Additionally, it can support the use of evidence within clinical practice to ultimately improve core patient outcomes.

## FUNDING INFORMATION

There was no funding associated with this work.

## CONFLICT OF INTEREST STATEMENT

No conflict of interest has been declared by the author(s) in relation to this study.

## ETHICS STATEMENT

This QI project was registered and approved within the Trust's governance system and allocated a reference number ID 9562. All staff were notified that survey responses may be used for publication and were given the option to have their data removed from the analysis. No participants withdrew their data.

## PATIENT CONSENT STATEMENT

No patient consent was required for this work.

## Supporting information


**Data S1.** Supporting information.

## Data Availability

The data that support the findings of this study are available from the corresponding author upon reasonable request.
