# Supplementary material for: Tea trolley teaching in critical care: Integrating evidence‐based practice with library services
Source: Nurs Crit Care. 2025 Feb 2;30(5):e13264. doi: 10.1111/nicc.13264 (PMC12332469; doi:10.1111/nicc.13264)
Supplement: Supplementary file 1 — Data S1. Supporting information. [file NICC-30-0-s001.docx]

## SQUIRE Reporting checklist

Revised Standards for Quality Improvement Reporting Excellence

SQUIRE 2.0

| Notes to Authors | | Page number |
| --- | --- | --- |
| - The SQUIRE guidelines provide a framework for reporting new knowledge about how to improve healthcare. - The SQUIRE guidelines are intended for reports that describe [system](http://www.squire-statement.org/index.cfm?fuseaction=page.viewpage&pageid=485#System) level work to improve the quality, safety, and value of healthcare, and used methods to establish that observed outcomes were due to the [intervention(s).](http://www.squire-statement.org/index.cfm?fuseaction=page.viewpage&pageid=485#Interventions) - A range of approaches exists for improving healthcare.  SQUIRE may be adapted for reporting any of these. - Authors should consider every SQUIRE item, but it may be inappropriate or unnecessary to include every SQUIRE element in a particular manuscript. - The SQUIRE Glossary contains definitions of many of the key words in SQUIRE. - The [Explanation and Elaboration](http://www.squire-statement.org/index.cfm?fuseaction=page.viewpage&pageid=504)document provides specific examples of well-written SQUIRE items, and an in-depth explanation of each item. - Please cite SQUIRE when it is used to write a manuscript. | |  |
| Title and Abstract | |  |
| **1.  Title** | Indicate that the manuscript concerns an [initiative](http://www.squire-statement.org/index.cfm?fuseaction=page.viewpage&pageid=485#Initiative) to improve healthcare (broadly defined to include the quality, safety, effectiveness, patient-centeredness, timeliness, cost, efficiency, and equity of healthcare) | [Abstract,](#_Adaptation_of_an) Title page |
| **2.  Abstract** | a.  Provide adequate information to aid in searching and indexing  b.  Summarize all key information from various sections of the text using the abstract format of the intended publication or a structured summary such as: background, local [problem](http://www.squire-statement.org/index.cfm?fuseaction=page.viewpage&pageid=485#Problem), methods, interventions, results, conclusions | Pg 1  Pg 1 |
| Introduction | *Why did you start?* |  |
| [**3. Problem Description**](http://www.squire-statement.org/index.cfm?fuseaction=page.viewpage&pageid=485#Problem) | Nature and significance of the local [problem](http://www.squire-statement.org/index.cfm?fuseaction=page.viewpage&pageid=485#Problem) | Pg 1 |
| **4. Available Knowledge** | Summary of what is currently known about the [problem](http://www.squire-statement.org/index.cfm?fuseaction=page.viewpage&pageid=485#Problem), including relevant previous studies | Pg 1 |
| **5. Rationale** | Informal or formal frameworks, models, concepts, and/or [theories](http://www.squire-statement.org/index.cfm?fuseaction=page.viewpage&pageid=485#Theory) used to explain the [problem](http://www.squire-statement.org/index.cfm?fuseaction=page.viewpage&pageid=485#Problem), any reasons or [assumptions](http://squire.citysoft.org/index.cfm?fuseaction=page.viewPage&pageID=485&nodeID=1#assumptions) that were used to develop the [intervention(s),](http://www.squire-statement.org/index.cfm?fuseaction=page.viewpage&pageid=485#Interventions)and reasons why the [intervention(s)](http://www.squire-statement.org/index.cfm?fuseaction=page.viewpage&pageid=485#Interventions) was expected to work | Pg 1-2 |
| **6. Specific Aims** | Purpose of the project and of this report | [Pg](#_Aim) 2 |
| Methods | *What did you do?* |  |
| [**7. Context**](http://www.squire-statement.org/index.cfm?fuseaction=page.viewpage&pageid=485#context) | Contextual elements considered important at the outset of introducing the [intervention(s)](http://www.squire-statement.org/index.cfm?fuseaction=page.viewpage&pageid=485#Interventions) | Pg 2 |
| [**8. Intervention(s)**](http://www.squire-statement.org/index.cfm?fuseaction=page.viewpage&pageid=485#Interventions) | a.  Description of the [intervention(s)](http://www.squire-statement.org/index.cfm?fuseaction=page.viewpage&pageid=485#Interventions) in sufficient detail that others could reproduce it  b.  Specifics of the team involved in the work | Pg 2 |
| **9. Study of the Intervention(s)** | a.  Approach chosen for assessing the impact of the [intervention(s)](http://www.squire-statement.org/index.cfm?fuseaction=page.viewpage&pageid=485#Interventions)  b.  Approach used to establish whether the observed outcomes were due to the [intervention(s)](http://www.squire-statement.org/index.cfm?fuseaction=page.viewpage&pageid=485#Interventions) | Pg 3 |
| **10. Measures** | a.  Measures chosen for studying [processes](http://www.squire-statement.org/index.cfm?fuseaction=page.viewpage&pageid=485#Process) and outcomes of the [intervention(s),](http://www.squire-statement.org/index.cfm?fuseaction=page.viewpage&pageid=485#Interventions)including rationale for choosing them, their operational definitions, and their validity and reliability  b.  Description of the approach to the ongoing assessment of contextual elements that contributed to the success, failure, efficiency, and cost  c.  Methods employed for assessing completeness and accuracy of data | [Pg](#_Preliminary_Data) 3 |
| **11. Analysis** | a.  Qualitative and quantitative methods used to draw [inferences](http://www.squire-statement.org/index.cfm?fuseaction=page.viewpage&pageid=485#Inferences) from the data  b.  Methods for understanding variation within the data, including the effects of time as a variable | [Pg](#_Preliminary_Data) 3-4 |
| **12. Ethical Considerations** | [Ethical aspects](http://www.squire-statement.org/index.cfm?fuseaction=page.viewpage&pageid=485#Ethical_aspects)of implementing and studying the [intervention(s)](http://www.squire-statement.org/index.cfm?fuseaction=page.viewpage&pageid=485#Interventions) and how they were addressed, including, but not limited to, formal ethics review and potential conflict(s) of interest | Pg 5, Ethics statement |
| Results | *What did you find?* |  |
| **13. Results** | a.  Initial steps of the [intervention(s)](http://www.squire-statement.org/index.cfm?fuseaction=page.viewpage&pageid=485#Interventions) and their evolution over time (e.g., time-line diagram, flow chart, or table), including modifications made to the intervention during the project  b.  Details of the [process](http://www.squire-statement.org/index.cfm?fuseaction=page.viewpage&pageid=485#Process) measures and outcome  c.  Contextual elements that interacted with the [intervention(s)](http://www.squire-statement.org/index.cfm?fuseaction=page.viewpage&pageid=485#Interventions)  d.  Observed associations between outcomes, interventions, and relevant contextual elements  e.  Unintended consequences such as unexpected benefits, [problems](http://www.squire-statement.org/index.cfm?fuseaction=page.viewpage&pageid=485#Problem), failures, or costs associated with the [intervention(s).](http://www.squire-statement.org/index.cfm?fuseaction=page.viewpage&pageid=485#Interventions)  f.  Details about missing data | Pg 3-4  Pg 3-4  n/a  n/a  n/a  n/a |
| Discussion | *What does it mean?* |  |
| **14. Summary** | a.  Key findings, including relevance to the [rationale](http://www.squire-statement.org/index.cfm?fuseaction=page.viewpage&pageid=485#Rationale) and specific aims  b.  Particular strengths of the project | [Pg](#_Summary) 4 |
| **15. Interpretation** | a.  Nature of the association between the [intervention(s)](http://www.squire-statement.org/index.cfm?fuseaction=page.viewpage&pageid=485#Interventions) and the outcomes  b.  Comparison of results with findings from other publications  c.  Impact of the project on people and [systems](http://www.squire-statement.org/index.cfm?fuseaction=page.viewpage&pageid=485#Systems)  d.  Reasons for any differences between observed and anticipated outcomes, including the influence of [context](http://www.squire-statement.org/index.cfm?fuseaction=page.viewpage&pageid=485#context)  e.  Costs and strategic trade-offs, including [opportunity costs](http://www.squire-statement.org/index.cfm?fuseaction=page.viewpage&pageid=485#Opportunity_costs) | [Pg](#_Summary) 4 |
| **16. Limitations** | a.  Limits to the [generalizability](http://www.squire-statement.org/index.cfm?fuseaction=page.viewpage&pageid=485#Generalizability) of the work  b.  Factors that might have limited [internal validity](http://www.squire-statement.org/index.cfm?fuseaction=page.viewpage&pageid=485#Internal_validity) such as confounding, bias, or imprecision in the design, methods, measurement, or analysis  c.  Efforts made to minimize and adjust for limitations | n/a |
| **17. Conclusions** | a.  Usefulness of the work  b.  Sustainability  c.  Potential for spread to other [contexts](http://www.squire-statement.org/index.cfm?fuseaction=page.viewpage&pageid=485#context)  d.  Implications for practice and for further study in the field  e.  Suggested next steps | [Pg](#_Summary) 4 |
| Other Information |  |  |
| **18. Funding** | Sources of funding that supported this work. Role, if any, of the funding organization in the design, implementation, interpretation, and reporting | Pg 5, Funding statement |
